# Synergistic Enhancement of Capacitive Performance in Porous Carbon by Phenolic Resin and Boric Acid

**DOI:** 10.3390/molecules30061228

**Published:** 2025-03-09

**Authors:** Yingkai Xia, Fengzhi Zhang, Shuo Wang, Shuang Wei, Xu Zhang, Wei Dong, Ding Shen, Shuwei Tang, Fengxia Liu, Yuehui Chen, Shaobin Yang

**Affiliations:** 1 School of Mining, Liaoning Technical University, Fuxin 123000, China; xiayingkai200719@126.com (Y.X.); weishuangcoyi@163.com (S.W.); 2 Chengxi (Taizhou) Equipment Technology Co., Ltd., Jingjiang 214500, China; jameszhang_2019@163.com; 3 College of Material Science and Engineering, Liaoning Technical University, Fuxin 123000, China; 19824844653@163.com (S.W.); liufengxia1981@126.com (F.L.); zhangxu@lntu.edu.cn (X.Z.); lgddongwei@163.com (W.D.); shending@lntu.edu.cn (D.S.); tangsw911@nenu.edu.cn (S.T.); 4 College of Science, Liaoning Technical University, Fuxin 123000, China; chenyuehui@lntu.edu.cn

**Keywords:** porous carbon electrode materials, boric acid, phenol-formaldehyde resin, lithium-ion capacitors, ultramicropores and small mesopores

## Abstract

The study of pore structure regulation methods has always been a central focus in enhancing the capacitance performance of porous carbon electrodes in lithium-ion capacitors (LICs). This study proposes a novel approach for the synergistic regulation of the pore structure in porous carbon using phenol-formaldehyde (PF) resin and boric acid (BA). PF and BA are initially dissolved and adsorbed onto porous carbon, followed by hydrothermal treatment and subsequent heat treatment in a N_2_ atmosphere to obtain the porous carbon materials. The results reveal that adding BA alone has almost no influence on the pore structure, whereas adding PF alone significantly increases the micropores. Furthermore, the simultaneous addition of PF and BA demonstrates a clear synergistic effect. The CO_2_ and H_2_O released during the PF pyrolysis contribute to the development of ultramicropores. At the same time, BA facilitates the N_2_ activation reaction of carbon, enlarging the small mesopores and aiding their transformation into bottlenecked structures. The resulting porous carbon demonstrates an impressive capacitance of 144 F·g^−1^ at 1 A·g^−1^ and a capacity retention of 19.44% at 20 A·g^−1^. This mechanism of B-catalyzed N_2_-enhanced mesopore formation provides a new avenue for preparing porous carbon materials. This type of porous carbon exhibits promising potential for applications in Li-S battery cathode materials and as catalyst supports.

## 1. Introduction

Lithium-ion capacitors (LICs) offer the significant advantage of high power compared with lithium-ion batteries, which has garnered widespread attention [1,2]. However, due to their limited capacity and poor performance at high currents, they have not yet reached commercial application stages [3,4]. Therefore, improving capacitance is one of the key challenges in LIC research.

Carbon materials are commonly employed as electrode components in LICs due to their high specific surface area, structural stability, and excellent corrosion resistance [5,6]. In capacitors, carbon electrode materials primarily store energy through the adsorption of electrolyte ions onto the electrode surfaces, forming a double electric layer. The pore structure of porous carbon electrodes is closely related to their capacitance performance. Early studies indicated that a high specific surface area helps increase the adsorption capacity, enhancing capacitance performance [7,8]. In 2006, Gogist et al. [9] discovered that ultramicropores smaller than 1 nm enable the desolvation of electrolyte ions, significantly improving capacitance. However, subsequent studies using infrared methods to measure the ion diffusion rates in different pore sizes found that ion diffusion is slower in micropores, reducing the high-current performance of the material. In contrast, ions diffuse more quickly in small mesopores (2–5 nm), leading to better high-current performance [10,11]. Therefore, increasing the proportion of mesopores in microporous carbon materials and enhancing ion-transport pathways are essential for improving capacitance.

The primary preparation methods for carbon electrode materials include activation [12,13], vapor deposition [14], Metal–Organic Framework (MOF) [15], and templating [16]. Chemical activation typically involves increasing the concentration of activating agents such as KOH, ZnCl_2_, and H_3_PO_4_ and raising the temperature. This process significantly increases the content of ultramicropores in the resulting carbon materials, but the pore distribution is not well controlled [17]. Conversely, physical activation using agents like CO_2_ or H_2_O yields a high content of micropores within the range of 0.8–2 nm [18]. Vapor deposition can significantly increase the presence of pores smaller than 0.7 nm, but its effect on mesopores is limited [14]. Pores with diameters smaller than 0.7 nm are typically referred to as ultramicropores [19]. On the other hand, MOF and templating methods require post-template removal processes, which can introduce impurities and raise production costs [15]. Therefore, there is a pressing need for a method that can effectively enhance micropores and mesopores in porous carbon electrode materials.

This study used coal-based activated carbon (AC) as the precursor, introducing PF and BA. Following hydrothermal treatment and subsequent heat treatment in a N_2_ atmosphere, a carbon material with enhanced supermicropore and small mesopore content was obtained. This carbon material exhibited excellent electrochemical performance when applied to lithium-ion capacitors. Furthermore, the mechanism of pore structure regulation was analyzed. The regulation mechanism plays a critical role in the efficient and convenient production of carbon materials comprising micropores and mesopores.

## 2. Results and Discussion

This study initially introduced PF and BA separately into the porous carbon material to identify their individual effects on the regulation of the pore structure. The structural characterization of AC–BA is shown in Supplementary Material S1. Subsequently, both BA and PF were simultaneously introduced into the porous carbon, utilizing a hydrothermal heat-treatment method to achieve the incorporation of B. The mechanism by which BA and PF regulate the pore structure of the porous carbon was then further analyzed.

### 2.1. Impact of PF Modification

#### 2.1.1. Structural Analysis of PF Modification

**Figure 1:**
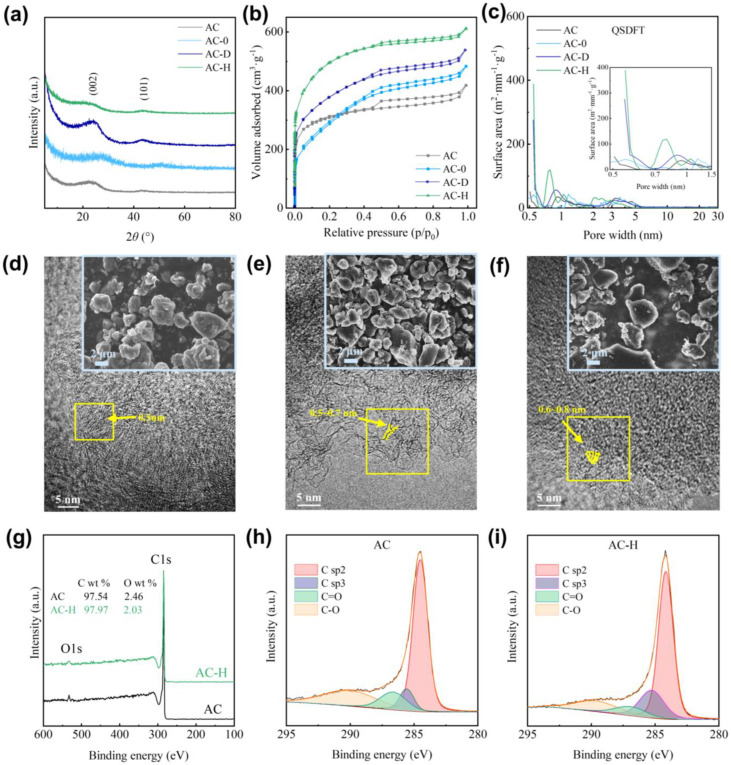
Phase and structural analysis of coal-based porous carbon samples: (**a**) XRD; (**b**) adsorption isotherms; (**c**) pore distribution; (**d**) microscopic morphology of AC; (**e**) microscopic morphology of AC-0; (**f**) microscopic morphology of AC-H; (**g**) XPS spectra of AC and AC-H; (**h**) C1s peak of AC; (**i**) C1s peak of AC-H.

X-ray diffraction (XRD) analysis was performed on the experimental activated carbon materials AC, AC-0, and AC-H to further understand the structural characteristics of the coal-based porous carbon samples obtained through hydrothermal carbon deposition with PF. Figure 1a presents the XRD results. All three samples exhibit broad diffraction peaks in the 20–25.6° and 41–42° ranges, indicating that the primary component of the samples is amorphous carbon, which contains a substantial amount of graphite-like carbon crystallites. The interlayer distance d_002_ corresponding to the (002) plane was calculated using Bragg’s law to characterize the carbon layer spacing, as shown in Table 1 [20]. It can be observed from the table that the d_002_ of AC was 0.3789 nm, while the d_002_ of AC-0 significantly decreased.

On the other hand, the d_002_ of AC-H increased to 0.3932 nm. This suggests that a small amount of PF was adsorbed onto the AC during the impregnation process. As the material undergoes heat treatment and interacts with the pore walls of AC, the d_002_ gradually increases. This phenomenon leads to the broadening of the diffraction peak associated with the (002) plane in the 20~25.6° range, further contributing to the amorphousness of the samples.

To investigate the effect of PF on the pore structure of the coal-based porous carbon, isothermal nitrogen adsorption tests were performed at 77 K on the coal-based porous carbon samples. Figure 1b illustrates the resulting adsorption curves. All curves correspond to type IVA, indicating the presence of both micropores and mesopores in these samples [21]. A hysteresis loop is observed between P/P_0_ = 0.35~0.95, with symmetric loops belonging to type H4, suggesting that the coal-based porous carbon samples contain slit-like pore structures within the mesopore region. Compared with AC, both AC-0 and AC-H samples demonstrate increased adsorption amounts, indicating a higher pore volume.

To further analyze the adsorption curves, the slit/cylindr pores@QSDFT model yielded the most accurate treatment of the nitrogen adsorption curves, indicating that the internal pore structure of the samples mainly consists of slit-like, plate-like pores. Following QSDFT analysis, the pore size distribution curves for the coal-based porous carbon samples were obtained, as illustrated in Figure 1c. The pore distribution for AC is mainly concentrated in the ranges of 0.5–0.65 nm, 0.65–2 nm, and 2–5.5 nm. After PF modification, the pore size distribution of AC-H remains concentrated within these ranges, with a significant increase in pores in the 0.5–0.65 nm range. Although there is a slight decrease in pore sizes in the 0.65–2 nm and 2–5.5 nm ranges, the total pore area increases. In contrast, the pore distribution of AC-0 shows minimal change compared with the unmodified AC. This indicates that even a small amount of PF modification can significantly enhance the supermicropore content in porous carbon.

Table 1 presents the specific surface area and pore volume of all the coal-based porous carbon samples. The surface area and pore volume of the AC-H are significantly higher than those of AC and AC-0, with SSA_DFT_ of 1699 m^2^·g⁻^1^ and V_DFT_ of 0.862 cm^3^·g⁻^1^. The large surface area improves the Li-ion storage performance of carbon electrode materials.

#### 2.1.2. Microstructural Morphology Analysis of PF Modification

To further investigate the pore structure of the coal-based porous carbon, the microstructures of AC, AC-0, and AC-H are shown in Figure 1d–f. The SEM images in the figures reveal that all three samples consist of irregular small particles with smooth edges. Notably, the particle diameter of the AC-H sample is slightly larger, which may be attributed to the bonding effect of PF. The TEM images reveal that the edges of the carbon layers are characterized by numerous pores, forming a curved, cage-like structure. This aligns with the slit/cylindr pores@QSDFT model used for pore distribution analysis. The pore size of AC is mainly concentrated between 0.50 and 0.70 nm, while AC-H shows an increased pore content, with the pore size primarily concentrated between 0.55 and 0.80 nm. This result is consistent with the pore distribution analysis, indicating that PF modification increases the supermicropore content and enlarges the pore size in the carbon material.

#### 2.1.3. Surface Properties Analysis of PF Modification

Figure 1g presents the XPS spectra of the characteristic samples, AC and AC-H, to investigate the surface properties of the coal-based porous carbon samples before and after hydrothermal heat treatment. Both samples exhibit characteristic peaks at binding energies of 284.1 eV and 532 eV, corresponding to the C1s and O1s peaks, respectively [22,23]. The figure reveals that AC and AC-H primarily consist of carbon, with only small amounts of oxygen. Notably, AC-H displays a slightly higher C content compared with AC, indicating that the incorporation of the phenol-formaldehyde resin reduces the oxygen content on the surface of the carbon material. The C1s peak deconvolution spectra for both materials are shown in Figure 1h,i. The spectra reveal that the types of carbon atoms in both samples are similar, with only minor variations in their relative content [22].

The hydrothermal heat treatment of PF significantly enhances the specific surface area of the coal-based porous carbon. The pore structure is adjusted with minimal change to the surface properties of the carbon material. This provides the experimental foundation for subsequent combined modification with BA and PF.

#### 2.1.4. Electrochemical Performance Analysis of PF Modification

**Figure 2:**
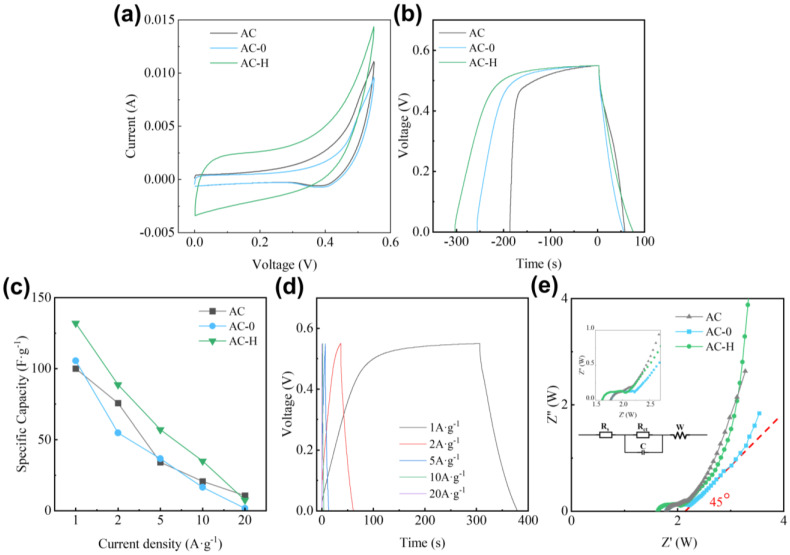
Capacitive performance of AC, AC-0, and AC-H samples. (**a**) CV curve at 10 mV·s^−1^; (**b**) GCD curves of three samples under 1 A·g^−1^; (**c**) the magnification performance of three samples; (**d**) GCD curves of AC-H under different current densities; (**e**) EIS performance curves of three samples.

Cyclic voltammetry (CV) tests were performed at a sweep rate of 10 mV·s^−1^, with results illustrated in Figure 2a, to evaluate the electrochemical performance of coal-based porous carbon materials. The AC and AC-0 samples exhibit oxidation peaks in the range of 0.38–0.44 V and reduction peaks around 0.5 V, which might be attributed to the ash content in the AC. In contrast, the CV curve of the PF-modified AC-H sample demonstrates improved symmetry, suggesting that incorporating PF enhances the Li-ion double-layer energy storage capabilities.

To evaluate the charge–discharge performance, galvanostatic charge–discharge (GCD) tests were conducted on the coal-based porous carbon samples at a current density of 1 A·g^−1^, with the results presented in Figure 2b. The GCD curves for AC, AC-0, and AC-H exhibit poor symmetry, characterized by extended charge platforms observed in the 0.5~0.55 V range, corresponding to the peaks at 0.5–0.55 V in the CV curves. The discharge platforms between 0.25 and 0.4 V correspond to the peaks at 0.38~0.44 V in the CV curves. Notably, the PF-modified AC-H exhibits a capacitance of 132 F·g^−1^ at a current density of 1 A·g^−1^, higher than that of AC, which has a capacitance of 100 F·g^−1^. Further analysis reveals that PF modification reduces charge–discharge efficiency. At a current density of 1 A·g^−1^, the charge–discharge efficiency of AC is 28.86%, whereas AC-H shows a lower efficiency of 23.20%, which is significantly below the efficiency of AC (28.86%).

The GCD tests were conducted at varying current densities to evaluate the capacitive performance of the three samples, with the results illustrated in Figure 2c. As the current density increases, a decline in specific capacitance is observed. At 10 A·g^−1^, the PF-modified sample AC-H maintains a specific capacitance of 35.02 F·g^−1^, corresponding to a retention rate of 26.53%. However, when the current density is further increased to 20 A·g^−1^, the capacity retention quickly decreases to 8.12%. The GCD curves of AC-H at different current densities are presented in Figure 2d, indicating that while the symmetry of the GCD curves improves with increasing current density, the capacity decreases.

To evaluate the charge transfer and electrolyte diffusion resistance in the coal-based activated carbon electrode materials, EIS tests were performed on all three samples at open-circuit potential over a frequency range of 1 to 10^5^ Hz, with the Nyquist plots illustrated in Figure 2e. The Nyquist curves for all three samples exhibit a dual structure: a semicircle in the high-frequency region and a straight line in the low-frequency region [24,25,26]. In the high-frequency region, the left side of the semicircle determines the electrode surface resistance [24,25]. Following PF modification, there is a slight decrease in the surface resistance of the material, likely due to a reduction in surface oxygen content. On the other hand, the radius of the semicircle in the high-frequency region of the Nyquist plot for AC-H increases, indicating a minimal diffusion resistance, which may be attributed to the increased micropore content in AC-H [25,27]. In the low-frequency region, the straight-line slope corresponds to the resistance to the diffusion of electrolyte ions toward the electrode surface (referred to as Warburg impedance). As illustrated in the figure, the straight lines for the AC-H sample in the low-frequency region approach a 45° angle, indicating improved diffusion performance after modification.

In summary, among the three coal-based porous carbons, AC-H as an electrode material is more advantageous for electrochemical applications.

### 2.2. Impact of BA and PF Co-Modification

#### 2.2.1. Structural Analysis of Co-Modification

**Figure 3:**
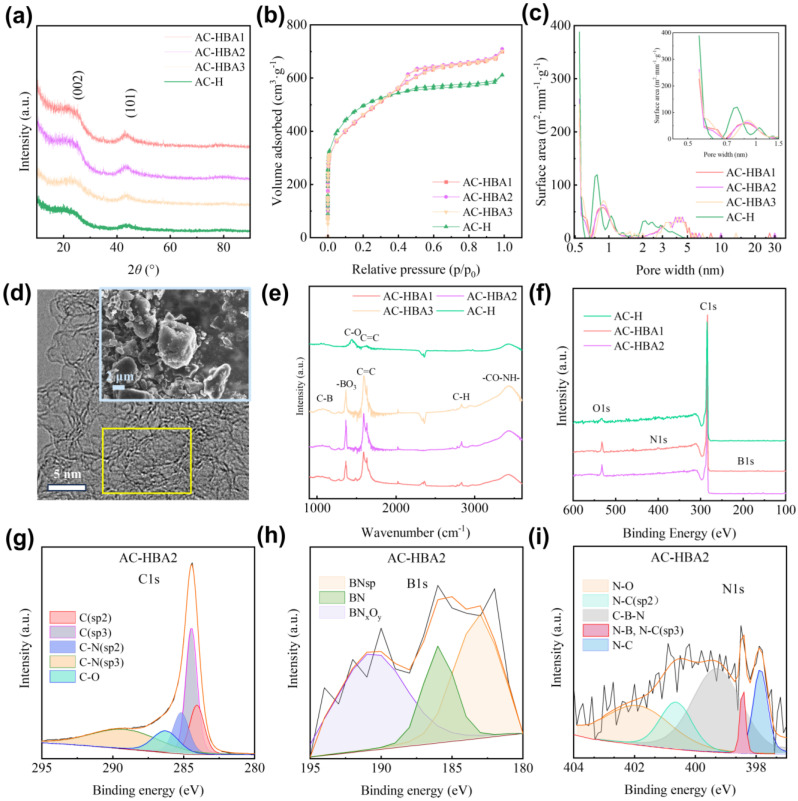
Phase and structural analysis of AC-H and co-modification samples: (**a**) XRD; (**b**) adsorption curve; (**c**) pore distribution; (**d**) microstructure of AC-HBA2; (**e**) FTIR; (**f**) XPS curves of AC-H, AC-HBA1, and AC-HBA2; (**g**) C1s peak of AC-HBA2; (**h**) B1s peak of AC-HBA2; (**i**) N1s peak of AC-HBA2.

To gain a deeper understanding of the structure after co-modification with BA and PF, the XRD analysis was performed on the AC-H and the co-modified samples, as illustrated in Figure 3a. The results indicate that the three co-modified samples exhibit similar characteristics to AC-H, with broadened diffraction peaks appearing between 20 to 25.6° and 40 to 44°. This suggests that the main component of the sample is amorphous carbon. Due to the low concentration of BA used for doping, no distinct B-containing phases were detected in the XRD pattern. The interlayer spacing d_002_ corresponding to the (002) diffraction peak was calculated to characterize the carbon layer spacing, as shown in Table 2 [21]. Notably, with an increase in BA concentration, the d002 of the co-modified samples slightly increased, with the d002 for the AC-HBA2 sample being the largest, at 0.3764 nm.

To evaluate the pore structure of these samples, nitrogen adsorption isotherms were measured at 77 K, as demonstrated in Figure 3b. The isotherms for the three co-modified samples exhibit striking similarities, all conforming to type IB isotherms. A small amount of adsorption in the low-pressure region (P/P_0_ < 0.1) implies the presence of micropores within the samples [22]. However, unlike AC-H, the co-modified samples reveal a hysteresis loop in the P/P_0_ range of 0.4–0.6, resembling the H2 type and indicating the presence of bottle-neck-like mesopores [22].

Further analysis of the adsorption curves utilizing the QSDFT model enabled the determination of the pore size distribution, as illustrated in Figure 3c. The pore distribution in the BA and PF co-modified samples closely resembles that of the PF-modified AC-H. The pores are primarily concentrated in three ranges: 0.50~0.60 nm, 0.80~1.50 nm, and 2.5~5.5 nm. Following co-modification, the micropores in the 0.50~0.60 nm and 0.80~1.50 nm ranges slightly decreased, while the small mesopores in the 2.5~5.5 nm range significantly increased. The pore structure parameters for the samples are listed in Table 2. The trends for specific surface area (SSA_BET_) obtained via the traditional BET method align consistently with the surface area (SSA_DFT_) results. The V_DFT_ for the co-modified samples significantly increased. As the BA content decreased, the SSA_DFT_ slightly increased, and the pores around 0.7 nm became more prevalent. The AC-HBA2 sample exhibited the highest SSA_DFT_ and V_DFT_, measuring 1640 m^2^·g^−1^ and 1.023 cm^3^·g^−1^, respectively.

#### 2.2.2. Microscopic Morphology Analysis of Co-Modification

To conduct a more thorough analysis of the microstructure of the samples after co-modification, both SEM and TEM were utilized for observation. Figure 3d illustrates the microscopic morphology of AC-HBA2 after co-modification. The sample exhibits irregular small particles with relatively smooth edges, similar to the morphology of AC-H. The TEM images reveal numerous pores at the edges of the carbon layers, forming a curved, cage-like structure. The observations align with the slit/cylindr pores@QSDFT model analysis. 

#### 2.2.3. Surface Property Analysis of Co-Modification

To analyze the surface functional group content of the co-modified samples, FTIR spectroscopy was employed to examine the AC-H and the three co-modified samples, as shown in Figure 3e. The AC-H primarily exhibits two characteristic peaks at 1630 cm^−1^ and 1450 cm^−1^, corresponding to the symmetric stretching vibrations of C-O and C=C, indicating that carbon is the main component of the material [28]. After co-modification, all samples display five characteristic peaks, with a significant enhancement at 3350 cm^−1^, attributed to the -CO-NH- group [29]. This enhancement results from the reaction of nitrogen (N_2_) at 800 °C within the coal-based porous carbon, confirming the successful incorporation of boron into the material [28]. The peak at 2930 cm^−1^ is caused by C-H, stemming from the interaction between carbon, water, and PF during the hydrothermal process. The peak at 1630 cm^−1^, corresponding to C=C, shows a noticeable enhancement following co-modification. Additionally, the peak at 1360 cm^−1^ is associated with the -BO_3_ group induced by BA, further confirming the successful incorporation of boron [28,30]. A minor increase in the peak near 1100 cm^−1^ is attributed to the formation of C-B, providing further evidence of the successful introduction of B [31].

To further investigate the elemental composition and content of the co-modified carbon materials, X-ray photoelectron spectroscopy (XPS) was employed to analyze samples AC-3/BA1, AC-3/BA2, and AC-H, with the results shown in Figure 3f. All three samples exhibit distinct C1s and O1s peaks. The oxygen contents in AC-3/BA1 and AC-3/BA2 are 5.72% and 4.56%, respectively, enhancing the O1s signal in the co-modified samples. This enhancement may be attributed to the formation of B_2_O_3_ during the heat treatment, which significantly limits the amount of boric acid (BA) that can be incorporated during modification [32]. The B1s and N1s peaks are less pronounced in AC-3/BA1 and AC-3/BA2, with a nitrogen content of 1.38% and 0.71%, respectively, while the boron content remains below 1%. This lower concentration is due to the low BA concentration in preparing AC-3/BA1 (with only 1% BA by mass in the AC-3/BA1 sample) [33].

To confirm the presence of B and N elements, a detailed analysis was performed on AC-3/BA2 as an example. The C1s peak within the 280~295 eV range is illustrated in Figure 3g. Following peak fitting, the peaks at 283.95 eV, 284.5 eV, 285.8 eV, 287 eV, and 288.9 eV correspond to Csp^2^, Csp^3^, C-N (sp^2^), C-N (sp^3^), and C-O, respectively [33,34]. The B1s peak in the 180~195 eV range is presented in Figure 3h, where the results of peak fitting indicate that the peaks at 183.2 eV, 186.3 eV, and 190.5 eV are associated with B~N (sp^2^), BN, and an intermediate BN_x_O_y_ phase transitioning from B_2_O_3_ to BN, respectively. This analysis confirms the presence of boron in the sample. During the experiment, boric acid was initially converted to B_2_O_3_, which subsequently transformed into BN_x_O_y_ and BN under N_2_ atmosphere, establishing bonds with carbon [34,35]. The N1s peak in the 397–404 eV range is illustrated in Figure 3i, in which peak fitting reveals that the peaks at 397.5 eV, 398.4 eV, 399.5 eV, 400.5 eV, and 402 eV correspond to N-C, N-B, N-C (sp^3^), C-B-N, N-C (sp^2^), and N-O, respectively. This further confirms the presence of N elements and their interaction with B, C, and O [34].

To further validate the presence of boron in the co-modified samples, an ICP test was performed on the AC-HBA2, revealing N and B contents of 0.071% and 0.05%, respectively. This indicates that N and B have been successfully incorporated into the material, which closely aligns with the 1% BA content introduced in the AC-HBA2 experiment. It is important to emphasize that while XPS measures the surface elemental content of the material, ICP assesses the actual elemental content throughout the sample. Therefore, the higher proportions of N and B observed via XPS compared with ICP further confirm their incorporation on the surface of the carbon material.

These tests indicate that the mixture of BA and PF followed by hydrothermal heat treatment can successfully introduce a high concentration of ultramicropores (0.5~0.6 nm) and small mesopores (3~5 nm) without compromising the pore structure. Additionally, BN_x_O_y_ and BN are formed on the surface of the carbon, providing new insights into the preparation of micropore–mesopore materials for energy storage, catalysis, and other applications.

#### 2.2.4. Capacitance Performance Analysis of Co-Modification

**Figure 4:**
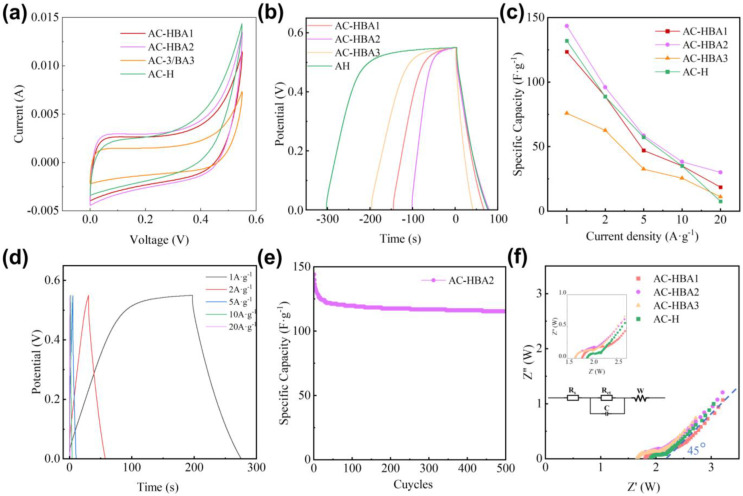
Capacitive performance of AC-H, AC-HBA1, AC-HBA2, and AC-HBA3. (**a**) CV curves at 10 mV·s^−1^; (**b**) GCD curves at 1 A·g^−1^; (**c**) rate performance; (**d**) GCD curves of AC-H at different current densities; (**e**) long-cycle performance of AC-HBA2; (**f**) EIS performance curves of these four samples.

To evaluate the electrochemical performance of BA and PF, CV tests were conducted on AC-H and the co-modified samples at a scan rate of 10 mV·s^−1^, as illustrated in Figure 4a. The CV curves for the co-modified samples exhibit better symmetry than AC-H, with the AC-HBA2 sample demonstrating the best symmetry, suggesting enhanced suitability for double-layer energy storage.

To evaluate the capacitance of the materials, GCD tests were performed on the co-modified samples at a current density of 1 A·g^−1^, as illustrated in Figure 4b. In contrast to the PF-modified AC-H, the co-modified samples exhibit significantly shorter charging plateaus, indicating improved charge–discharge performance. The capacitance values for AC-HBA1, AC-HBA2, and AC-HBA3 at 1 A·g^−1^ are 123 F·g^−1^, 144 F·g^−1^, and 98 F·g^−1^, respectively. The capacity performance of AC-HBA2 achieves the best levels reported in the literature. The capacity of porous carbon materials for LIC in the related literature [36,37,38,39,40,41] is provided in Supplementary Material S1. Notably, the charge–discharge efficiency of AC-HBA2 increased significantly, from 25.59% to 78.62%, revealing that the most significant enhancement in capacitance occurs at a BA-to-AC mass ratio of 0.1%. Conversely, when the BA mass ratio is decreased to 0.01%, the capacitance of AC-HBA3 experiences a considerable decline.

Additionally, AC-HBA1 exhibits a higher nitrogen content of 1.5% compared with AC-HBA2 (0.76%). Both values fall within the favorable range for N to enhance capacitance (0–7.4%) reported [35,42]. The capacitance and rate performance of AC-HBA1 are lower than those of AC-HBA2, suggesting that the small amounts of BN and BN_x_O_y_ incorporated into the carbon electrode material have a negligible effect on capacity. Instead, the primary factor contributing to the enhancement in capacity is the alteration in pore structure.

To evaluate the rate performance of the samples, GCD tests were conducted at varying current densities for the co-modified samples, with the results illustrated in Figure 4c. The co-modified samples exhibit superior capacity retention at a high current rate, especially at 20 A·g^−1^, compared with AC-H. Among the three samples, AC-HBA2 demonstrates the best performance at high current rates, achieving specific capacities of 38.24 F·g^−1^ and 28 F·g^−1^ at 10 A·g^−1^ and 20 A·g^−1^, respectively, along with capacity retention rates of 26.64% and 19.44%. A further examination of the GCD performance of AC-HBA2 across different current densities is presented in Figure 4d. As current density increases, the symmetry of the GCD curves improves, and the charge–discharge efficiency increases significantly. At a current density of 20 A·g⁻^1^, AC-HBA2 achieves a charge–discharge efficiency of 98.14%. Long-cycle tests conducted at 1 A·g⁻^1^ reveal that after 500 cycles, the capacity retention rate reaches 80.11%, as illustrated in Figure 4e.

To assess the charge transfer and electrolyte diffusion resistance in the co-modified electrode materials, electrochemical impedance spectroscopy (EIS) tests were conducted over a frequency range of 1 to 10^5^ Hz under open-circuit potential conditions, with the results illustrated in Figure 4f. The Nyquist curve reveals a semicircle in the high-frequency region and a straight line in the low-frequency region. In the low-frequency region, the straight line of AC-HBA2 approaches 45°, indicating that this sample facilitates the diffusion of the electrolyte within the electrode material [24,25,26]. It was observed that after BA doping, the diffusion coefficient of the samples increased, with AC-HBA2 reaching the highest value of 21.68 × 10^−16^. The method for calculating the diffusion coefficient is provided in Supplementary Material S2. In the high-frequency region, further analysis of the Nyquist curve facilitates the determination of the interface resistance (Rs) and charge transfer resistance (Rct). The semicircle in the high-frequency region corresponds to the Rs on the left side and the sum of the interface resistance Rs and Rct on the right side. The Rs represents the resistance the ions encounter in the electrolyte during their movement. The size of Rs in electrochemical tests directly affects current transmission and reaction rates. Rs, Rct, and the diffusion coefficient can be obtained by analyzing the Nyquist curve, as shown in Table 3 [24,26,27]. Comparatively, the Rs values for co-modified samples increased relative to AC-H, with AC-HBA1 exhibiting the highest Rs. This increase is likely due to the incorporation of BN_x_O_y_ and BN. Conversely, the Rct of co-modified samples demonstrated a slight decrease, indicating that the presence of boron aids in the diffusion of electrolyte ions within the electrode materials. Among the samples tested, AC-HBA2 displayed the lowest Rct, at 0.34 Ω, accompanied by the highest diffusion coefficient, which accounts for its superior electrochemical performance.

Introducing B and N elements into carbon electrode materials enhances capacitance and improves diffusion performance. However, this modification also increases surface resistance. Therefore, it is essential to introduce only a minimal amount of BA to effectively increase the capacitance. This is the fundamental rationale behind employing low concentrations of BA and PF to modify the carbon electrode materials in this study.

### 2.3. Synergistic Pore-Tuning Mechanism of PF and BA

**Figure 5:**
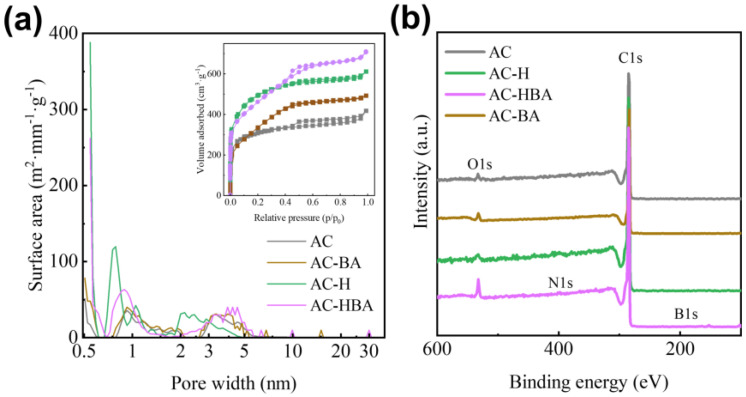
Influence of PF and BA on porous carbon structure: (**a**) pore distribution; (**b**) XPS.

To explore the pore-tuning mechanism facilitated by incorporating PF and BA into AC, the structure and surface properties of the following samples were analyzed: the raw coal-based activated carbon (AC), the BA-modified sample (AC-BA, with the phase characterization results provided in Supplementary Material S3), the PF-modified sample (AC-H), and the co-modified sample with BA and PF (AC-HBA). The results are presented in Figure 5.

**Figure 6:**
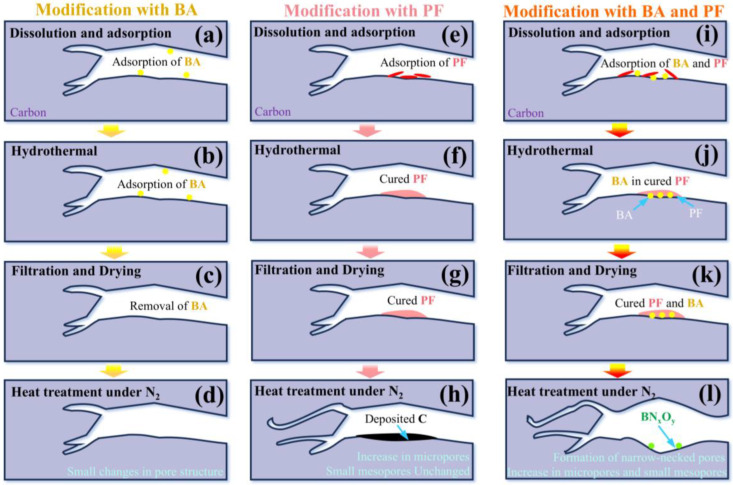
(**a**–**l**) Pore-tuning mechanism of PF and BA in porous carbon.

For the sample modified with BA alone, AC-BA, minimal changes were observed in the pore structure compared with AC (Figure 5a). Furthermore, XPS analysis (Figure 5b) revealed that no B1s or N1s peaks were detected in AC-BA, indicating that B was not successfully incorporated into the porous carbon. This lack of incorporation may be attributed to the fact that, while BA can enter the porous carbon with the solution and be adsorbed onto the pores (Figure 6a), it is removed during the washing process following the hydrothermal treatment (Figure 6c). Consequently, the effect of BA modification alone on the pore structure of the porous carbon is minimal (Figure 6d).

For the sample modified with PF alone, compared with AC, we found that the ultramicropores in AC-H significantly increased, particularly in the 0.54–0.6 nm and 0.7–0.9 nm ranges (Figure 6a). This finding indicates that PF modification is primarily responsible for enhancing the ultramicropores. PF can penetrate the porous carbon structure through the solution and be adsorbed into the pores (Figure 6e). PF undergoes crosslinking during the subsequent hydrothermal treatment, effectively solidifying the carbon pore walls, as Equation (1) outlines [43,44]. PF then undergoes carbonization in the following heat treatment process, as demonstrated in Equation (2), leading to carbon deposition on the pore walls. This process accounts for the observed decrease in the transition pore size of AC-H in Figure 6b. At elevated temperatures, the CO_2_ and H_2_ released from the PF decomposition are easily absorbed into ultramicropores, further reacting with the pore walls, as illustrated in Equations (3) and (4). This interaction etches the pore walls and increases the micropores by 0.54 nm. Additionally, the formed H_2_ reacts with the oxygen-containing functional groups on the surface of the porous carbon, reducing the O content in the carbon material [45,46]. This explains why the O content in AC-H is lower than that in AC (Figure 6b).

C_7_H_6_O_2_ → [-C_7_H_5_O-]_n_ + H_2_O (1)

[-C_7_H_5_O-]_n_ → C + CO_2_ + H_2_O (2)

H_2_O + C → CO + H_2_ (3)

CO_2_ + C → CO (4)

For the sample modified with both PF and BA, the pores of AC-HBA increased in the 0.5–0.6 nm, 0.8–1.5 nm, and 2.5–5.5 nm ranges (Figure 6a). This significant increase indicates that the synergistic effect of PF and BA increases both ultramicropores and small mesopores.

Both PF and BA can infiltrate the porous carbon structure through the solution, where they are adsorbed into the carbon pores (Figure 6i). During the hydrothermal treatment, PF undergoes crosslinking, and while solidifying, it encapsulates BA onto the pore walls, as illustrated in Figure 6j. The subsequent heat treatment leads to the carbonization of PF, akin to the outcomes observed with PF modification alone [43,44]. Concurrently, BA transforms into B_2_O_3_ during heat treatment, as shown in Equation (5) [32]. Figure 3d,e demonstrate that B_2_O_3_, in the presence of N_2_, further converts into BN and BN_x_O_y_, as described in Equations (6) and (7). Nitrogen oxides are released during this transformation, with NO being the predominant species at elevated temperatures [47]. These NO molecules further react with the carbon pore walls, releasing CO and N_2_. The reaction between B_2_O_3_ and carbon plays a catalytic role, facilitating the interaction of carbon with N_2_, which significantly reduces carbon near the B_2_O_3_, increasing the pore size [48]. This catalytic effect also contributes to the formation of bottleneck-shaped pores, corresponding to the H2-type hysteresis loop observed in the adsorption curve in Figure 2b. The synergistic pore-tuning mechanism of PF and BA is illustrated in Figure 6l.

H_3_BO_3_ → B_2_O_3_ + H_2_O (5)

B_2_O_3_ + N_2_ → BN + NO (6)

B_2_O_3_ + N_2_ → BN_x_O_y_ + NO (7)

NO + C → CO + N_2_ (8)

In summary, it was observed that using PF alone can significantly enhance the supermicroporosity of the carbon materials. However, when BA and PF are co-modified, PF effectively anchors boron onto the carbon pore surface. This interaction facilitates the catalytic activation of N_2_ and carbon reactions by boron, promoting the modulation of both ultramicropores and small mesopores. The synergistic effect of BA and PF in adjusting pore structure has been successfully demonstrated.

This study revealed that both PF modification alone and the co-modification of BA and PF improve capacitance. The increase in ultramicropores is advantageous for enhancing capacitance at low current densities. Moreover, the synergistic effect of BA and PF helps mitigate the rapid loss of capacity at high current densities, a common issue associated with PF modification alone. This observation implies that the presence of bottleneck-type transition pores provides a more effective channel for ion transport. The simultaneous enhancement of both ultramicropores and bottleneck-shaped small mesopores has resulted in improved capacitance performance. Furthermore, this work provides a novel approach for the industrial-scale production of carbon electrode materials containing a high density of ultramicropores and narrow-necked small mesopores.

## 3. Experiment

### 3.1. Materials

In this work, lean coal from Shanxi was uniformly mixed with KOH (China National Pharmaceutical Group, Beijing, China) at a mass ratio of 1:3. Under a nitrogen atmosphere, the mixture was kept at 800 °C for 2 h, washed, and dried to obtain coal-based porous carbon AC, which was used as the experimental raw material. PF and BA were sourced from China National Pharmaceutical Group. LiOH came from Shanghai Macklin Biochemical Technology Co., Ltd., Shanghai, China Ten grams of PF were dissolved in 1 L of deionized water to prepare PF solution for later use.

### 3.2. Preparation of Samples

#### 3.2.1. Modification with PF

PF was employed as the precursor for carbon deposition. AC was mixed with 101 mL of PF (mass of PF is 0.01 g) solution in 20 mL deionized water. The mixture underwent hydrothermal treatment at 160 °C for 2 h, followed by filtration and drying. Subsequently, the material was heat-treated at 800 °C for 2 h under a N_2_ atmosphere, and then cooled to room temperature in the furnace. After washing and drying, the sample was further ground and sieved to a particle size of less than 40 μm, resulting in the hydrothermally treated sample, AC-H. A mixture of AC and 20 mL of water was also treated by the same hydrothermal heat process, resulting in the AC-0 sample. The specific experimental conditions are detailed in Table 4. The samples were sealed and stored in a light-protected environment.

#### 3.2.2. Modification with BA

We modified AC by mixing 1 g of AC with 0.01 g of BA and 20 mL of water, followed by hydrothermal heat treatment, resulting in the AC-BA sample (Table 4).

#### 3.2.3. Modification with Both BA and PF

**Figure 7:**
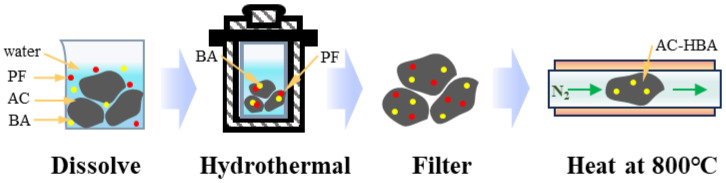
Hydrothermal heat treatment method of BA and PF.

Next, we performed a combined modification using both BA and PF. One gram of AC was mixed with 0.01 g of BA, 0.1 mL of PF solution, and 20 mL of water, followed by the hydrothermal heat treatment, resulting in the AC-HBA1 sample (Figure 7). Using similar methods, varying amounts of BA were introduced to produce the AC-HBA2 and AC-HBA3 samples (Table 4). These three samples are all coal-based porous carbons modified with BA and PF.

### 3.3. Analysis and Characterization

The X-ray diffraction analysis of the samples was conducted using a XRD-6100X (Shimadzu, Tokyo, Japan) diffractometer with a Cu target (λ = 0.154184 nm), a scan speed of 10°·min^−1^, a step size of 0.02°, and a diffraction angle range of 5° to 80°. Microstructural analysis was performed using scanning electron microscopy (SEM) and transmission electron microscopy (TEM) with a JSM-7500F and a JEM-2100F (JEOL, Beijing, China) instrument, respectively. Nitrogen adsorption analysis was conducted at 77 K using an AutosorbiQ (Anton Paar, Graz, Austria). Surface functional group analysis was performed using an IR Prestige-21 (Shimadzu, Tokyo, Japan) with KBr and a 250 (Thermo Fisher Escalab, 100 Technology Drive, Waltham, MA 02451, USA) X-ray photoelectron spectrometer with an Al Ka X-ray source (h = 1486.7 eV).

### 3.4. Electrochemical Performance Testing

A slurry was prepared by combining coal-based porous carbon (PC), a conductive agent (acetylene black), and a binder (polytetrafluoroethylene, PTFE) in a mass ratio of 8:1:1. This mixture was then uniformly coated onto a 1 cm × 1 cm piece of Ni foam, dried at 60 °C for 12 h, and subsequently compacted under a pressure of 4 MPa to fabricate a 1 cm^2^ square electrode.

LiOH was employed as the electrolyte, sourced from Shanghai Macklin Biochemical Technology Co., Ltd. The electrode was immersed in a 1 M solution of LiOH for 24 h prior to conducting electrochemical performance tests.

Electrochemical performance testing was executed using a three-electrode system. This system included a Hg/HgO electrode as the reference electrode, a 1 cm × 1 cm piece of Pt metal as the counter electrode, and a CHI660E electrochemical workstation to perform cyclic voltammetry (CV), galvanostatic charge–discharge (GCD), and electrochemical impedance spectroscopy (EIS). EIS measurements were executed at the open-circuit voltage over a frequency range from 1 to 10^5^ Hz.

## 4. Conclusions

This study presents a novel approach for the synergistic modulation of the pore structure in porous carbon using phenolic resin (PF) and boric acid (BA). Initially, PF and BA were dissolved and adsorbed onto porous carbon. A hydrothermal process was then employed to anchor BA onto the carbon pore walls through PF, followed by heat treatment in a N_2_ atmosphere to obtain porous carbon. The results indicate that adding BA alone does not significantly affect the pore structure. The incorporation of PF significantly increases the ultramicropores. However, the simultaneous addition of both PF and BA has a clear synergistic effect, leading to an increase in both ultramicropores and small mesopores. The mechanism for supermicropore formation is the activation of pore formation by the pyrolysis products of PF, while the mechanism for small mesopore formation involves BA catalysis. This process activates the carbon pore walls under N_2_ transforming plate-like mesopores into bottleneck-shaped pores. The resulting porous carbon demonstrates a high capacitance of 144 F·g^−1^ at 1 A·g^−1^, with a capacity retention of 19.44% at 20 A·g^−1^. The synergistic effect of BA and PF contributes to the development of porous carbon anode materials for Li-S batteries as well as catalytic support materials across various other applications.

## Figures and Tables

**Table 1:** Structural parameters of AC, AC-0, and AC-H.

Sample	*d*_002_ (g)	SSA_BET_ (m^2^·g^−1^)	SSA_DFT_ (m^2^·g^−1^)	SSA_0.5–1.5_ (m^2^·g^−1^)	SSA_2.5–5.5_ (m^2^·g^−1^)	V_DFT_ (cm^3^·g^−1^)	Average Pore Size (nm)	Minimum Pore Size (nm)
AC	0.3789	1026	914	394	3837	0.721	0.91	0.495
AC-0	0.3365	983	851	437	370	0.679	0.89	0.504
AC-H	0.3932	1729	1699	1212	263	0.862	0.54	0.545

**Table 2:** Structure parameters of the samples doped with PF and BA.

Sample	*d*_002_ (g)	SSA_BET_ (m^2^·g^−1^)	SSA_DFT_ (m^2^·g^−1^)	SSA_0.5–1.5_ (m^2^·g^−1^)	SSA_2.5–5.5_ (m^2^·g^−1^)	V_DFT_ (cm^3^·g^−1^)	Average Pore Size (nm)	Minimum Pore Size (nm)
AC-HBA1	0.3520	1610	1624	884	544	1.013	0.54	0.545
AC-HBA2	0.3764	1619	1640	901	545	1.023	0.54	0.545
AC-HBA3	0.3890	1620	1632	988	500	1.017	0.54	0.545
AC-H	0.3932	1729	1699	1212	263	0.862	0.54	0.545
AC	0.3789	1026	914	394	384	0.721	0.91	0.495

**Table 3:** Capacitive performance of the samples after BA and PF modification.

Sample	C_1A_ (F·g^−1^)	C_20A_/C_1A_ (%)	Rs (Ω)	Rct (Ω)	Diffusion Coefficient (10^−16^)
AC-HBA1	123	18.6	1.81	0.45	7.52
AC-HBA2	144	30.02	1.67	0.34	21.68
AC-HBA3	76	11.01	1.65	0.41	12.00
AC-H	132	7.44	1.13	0.67	6.66
AC	100	10.72	1.79	0.32	4.56

**Table 4:** Raw material ratios and preparation methods of the samples.

Samples	AC	PF	BA	H_2_O
AC-0	1 g	0	0	20 mL
AC-H	1 g	0.1 mL	0	20 mL
AC-BA	1 g	0	0.01 g	20 mL
AC-HBA1	1 g	0.1 mL	0.01 g	20 mL
AC-HBA2	1 g	0.1 mL	0.001 g	20 mL
AC-HBA3	1 g	0.1 mL	0.0001 g	20 mL

## Data Availability

All test data are provided in the manuscript and the supporting literature.

## Supplementary Materials

The following supporting information can be downloaded at: https://www.mdpi.com/article/10.3390/molecules30061228/s1, S1: Capacitive properties of porous carbon in this study versus references; S2: Diffusion coefficient; S3: Characterization of AC-BA samples.
