# Supplementary material for: Synergistic Enhancement of Capacitive Performance in Porous Carbon by Phenolic Resin and Boric Acid
Source: Molecules. 2025 Mar 9;30(6):1228. doi: 10.3390/molecules30061228 (PMC11946533; doi:10.3390/molecules30061228)
Supplement: Supplementary file 1 [file molecules-30-01228-s001.zip › molecules-3485340-supplementary.pdf]

SI Characterization of AC-BA samples

The characterization of the AC-BA sample, obtained after direct hydrothermal reaction using boric acid (BA) followed by heat treatment, is presented in Figure S1. Figure S1(a) shows the XRD curvrys, indicating that the main component is amorphous carbon composed of carbon microcrystallites, similar to AC-0. Figures S1(b) and (c) also indicate that its pore structure is analogous to that of AC-0. Both the FTIR analysis in Figure S1(d) and the XPS analysis in Figure S1(e) indicate that the sample does not contain the characteristic peaks of B and N elements, which suggests that using BA directly during the hydrothermal process and then heat treatment cannot successfully introduce B or N into carbon materials.

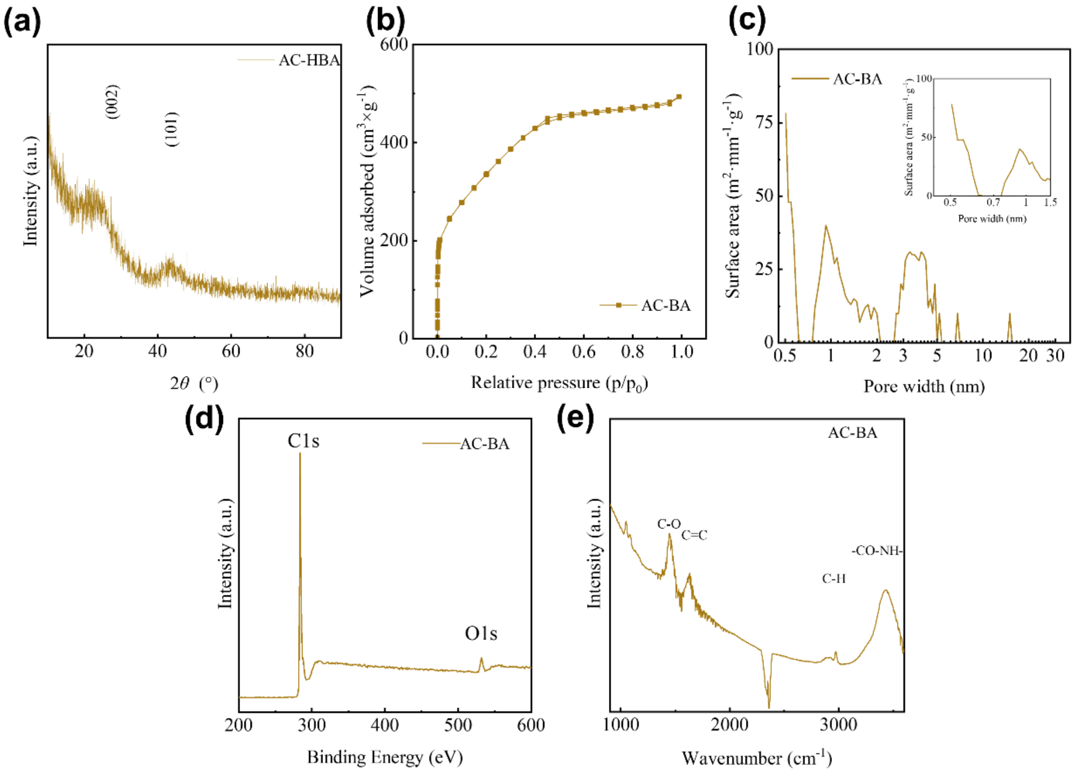

S2: Capacitive properties of porous carbon in this study versus references

Table S2 The capacitive performance of LIC carbon electrode material

Raw materials	Samples	SSA_{BET} ($m^2 \cdot g^{-1}$)	Electrolyte solution	Test methods	capacitance ($F \cdot g^{-1}$)	Reference
bituminous coal	AC-HBA2	1497	1M LiOH	Three-electrode system	164 ($1A \cdot g^{-1}$)	This work
coconut shell carbon	YP-50F (Kuraray)	1600	6M LiOH	Two-electrode system	113 ($0.9A \cdot g^{-1}$)	[1]
anthracite	YP-80F (Kuraray)	2100	6M LiOH	Two-electrode system	114 ($0.9A \cdot g^{-1}$)	[1]
PAF	MOLC	1084	LiOH	Two-electrode system	35 ($1A \cdot g^{-1}$)	[2]
polyaniline–phosphite	PANI-DPPH-C	<0.1	3M LiOH	Three-electrode system	57.6 ($5mV \cdot s^{-1}$)	[3]
activated carbon fiber	PP membrane	-	4.6M LiOH	Three -electrode Swagelok-type cells	108 ($1A \cdot g^{-1}$)	[4]
carbon black	SC3	1880	1M $LiPF_6$	Three -electrode Swagelok-type cells	115 ($5\ mA \cdot cm^{-2}$)	[5]
natural graphite	MWCNTs	<1	2M LiOH	Three -electrode Swagelok-type cells	40 ($1A \cdot g^{-1}$)	[6]

References

1. Karamanova, B.; Stoyanova, A.; Shipochka, M.; Veleva S.; Stoyanova R. Effect of alkaline-basic electrolytes on the capacitance performance of biomass-derived carbonaceous materials. *Materials* **2020**, *13*, 2941.
2. Shaibani, M.; Smith SJ, D.; Banerjee, P.C.; Konstas K.; Zafari A.; Lobo DE.; Nazari M; Hollenkamp A. F.; Hill M. R.; Majumder M. Framework-mediated synthesis of highly microporous onion-like carbon: Energy enhancement in supercapacitors without compromising power. *J. Mater. Chem. A* **2017**, *5*, 2519–2529.
3. Bober, P.; Trchová, M.; Morávková, Z.; Kovářová J; Vulić I.; Gavrilov N.; Pašti IA.; Stejskal J. Phosphorus and nitrogen-containing carbons obtained by the carbonization of conducting polyaniline complex with phosphites. *Electrochim. Acta* **2017**, *246*, 443–450.
4. Stepniak, I.; Ciszewski, A. New design of electric double layer capacitors with aqueous LiOH electrolyte as alternative to capacitor with KOH solution. *J. Power Sources* **2010**, *195*, 2564–2569.
5. Krause, A.; Kossyrev, P.; Oljaca, M.; Passerini S.; Winter M.; Balducci A. Electrochemical double layer capacitor and lithium-ion capacitor based on carbon black. *J. Power Sources* **2011**, *196*, 8836–8842.
